# Systematic Experimental Assessment of a 2D-Motion Sensor to Detect Relative Movement between Residual Limb and Prosthetic Socket

**DOI:** 10.3390/s18072170

**Published:** 2018-07-06

**Authors:** Veronika Noll, Stephan Rinderknecht, Philipp Beckerle

**Affiliations:** Institute for Mechatronic Systems in Mechanical Engineering, Technische Universität Darmstadt, Otto-Berndt-Str. 2, 64287 Darmstadt, Germany; rinderknecht@ims.tu-darmstadt.de (S.R.); beckerle@ims.tu-darmstadt.de (P.B.)

**Keywords:** relative movement, lower limb prosthetics, biomechanical measurement tasks, quantifying socket fit, gait analysis

## Abstract

A sensor system for measuring the relative movement between prosthetic socket and residual limb based on a 2D-motion sensor is introduced and thoroughly tested experimentally. The quantitative analysis of test rig evaluation is used to identify advantageous sensor settings and liner configurations. Considering these favorable settings, sensor functionality is quantified to $err_{rel}=0.52\pm 1.78$%. Advancing to convex measurement surfaces, the sensor shows absolute errors of $err_{abs}\leq 1$ mm in an observable measurement scenario. The feasibility of measuring gait-induced relative movement with the proposed 2D-motion sensor is shown via a biomechanical plausibility study. Overall, the findings suggest that the proposed sensor system is suitable for investigating the relative movement between residual limb and prosthetic socket in dynamic gait situations.

## 1. Introduction

Sockets are the mechanical interface which link the residual limb and the prosthetic device. While mechatronic concepts have improved the functionality of prostheses and thereby the quality of life of their users [1,2], socket designs are still mostly handcrafted. Thus, the fitting success is distinctly correlated to the ability and experience of the prosthetist, who examines the individuality of the residual limb in a static position [3]. Yet, the real interaction is dynamic due to the biomechanics of human gait, which might not be taken into account although they influence the well-being and mobility of amputees [4]. To overcome those issues, a quantitative assessment of the biomechanical interactions during gait appears very promising [4]. Most existing approaches rely on pressure measurements, computational modeling, and sensing friction-related phenomena, e.g., relative movement [4].

The coupling stiffness between residual limb and prosthetic socket is a key aspect of socket fitting and related to relative movement [5]. Orthopedic experts consider little relative movement as an indicator of good socket control [6], while skin irritation seems to be connected to the occurrence of relative movement [7]. However, measuring relative movement between residual limb and prosthesis is still a remarkable challenge and approaches suggested in the literature vary distinctly regarding geometrical definitions, measurement principles, and the considered load conditions. The most common techniques that are used to acquire relative movement data at the interface are: radiography [8,9,10,11,12,13,14,15,16], ultrasound [17,18,19], motion capture [20,21,22], and other optical means [23,24,25,26]. Recently, the correlation between fluctuations in vacuum pressure of elevated vacuum suspension systems and the amount of distal displacement have been investigated and shown to be correlated [27,28].

For different reasons, e.g., limitations to statics or altering of interface dynamics, these techniques have severe drawbacks. Thus, a novel measuring approach based on an optical 2D-motion sensor is proposed in [29]. Similar sensor types, which are mainly used in computer mice, have been described as suitable low-cost options for different measuring tasks [30,31,32,33,34,35,36,37]. Due to the reported high dependency of sensor functionality on measurement surroundings, e.g., measuring surface texture, the proposed sensor needs to be evaluated thoroughly.

This paper assesses the applicability of a 2D-motion sensor for measuring the relative movement between residual limb and prosthetic socket experimentally. (This will be referred to as biomechanical measuring task within the paper.) Section 2 introduces the sensor system based on the optical 2D-motion sensor ADNS-9800. For the experimental evaluation, two different approaches are used: test rig (details in Section 3) and gait evaluation (details in Section 4). Both strategies and the results are described and discussed. Finally, a conclusion about the suitability of the sensor for the proposed measuring task is drawn and an outlook on future work is given.

## 2. The Sensor System

This section describes the sensor system used to experimentally evaluate the applicability of the 2D-motion sensor for the described measurement task. The measuring concept itself has been proposed in [29], which gives a more detailed description of the electronic and software implementation.

### 2.1. Sensor System Design and Implementation

**Figure 1:**
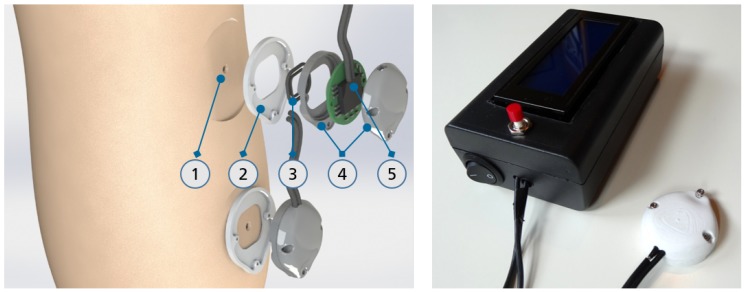
Exploded view of the sensor unit based on the 2D-motion sensor: **1** socket with clearance cavity, **2** mounting base, **3** seals, **4** sensor housing, and **5** sensor (**left**) as well as sensor system as a standalone, untethered, and integrated optical 2D-motion measuring system (**right**).

A breakout version (https://www.tindie.com/products/jkicklighter/adns-9800-optical-laser-sensor) of the ADNS 9800 from Pixart Inc. (Hsin-chu, Taiwan) is selected as optical motion sensor. The breakout version includes the ADNS 6190-lens from Pixart Inc. (Hsin-chu, Taiwan). This sensor is selected due to its all-over high-end specifications (cf. Section 2.2), compatibility (SPI-Interface), and availability in small quantities. The left picture in Figure 1 shows an exploded view of the sensor unit and its attachment to the socket via a mounting base that is glued to the socket wall. The socket wall needs a clearance cavity with a diameter of at least 2 mm, so that the optical sensor detects the surface of the residual limb. This cavity is the only structural change that is required to use the measuring system. Potential influences on the interaction between limb and socket is expected to be negligible due to the small diameter. Seals between the socket wall and the lens ensure that appropriate socket pressure conditions are maintained and that the lens is protected from dust and humidity. The sensor housing as well as mounting base is realized by a Fused-Deposition-Modeling 3D-printer with polyactide (PLA).

Figure 1 right shows a photograph of the sensor system. The sensor is integrated into the realized housing case and can be affixed to structures via three screws. The sensor is read out by a microcontroller (Arduino Uno, Arduino AG, Ivrea, Italy), which communicates with the sensor via SPI. The microcontroller is implemented into the shown black box which also includes a display to show online sensor data. Due to the included battery pack, the sensor system can be used as a standalone, untethered and integrated measuring system. Connecting the microcontroller to a personal computer via USB cable allows data storage and offline analysis in Matlab (MathWorks, Natick, MA, USA).

### 2.2. Sensor Unit Specifications

This section discusses the promoted sensor specifications in the data sheet with respect to the expected demand set by the planned biomechanical measuring task.

Commercially available gait analysis measurement systems use sampling rates above 50 Hz. The 2D-motion sensor has a programmable frame rate between 2000 and 12,000 fps. Depending on the realization of the measurement chain, a sufficient sampling rate is achieved. In the proposed sensor system, the sensor unit communicates via SPI with the microcontroller (Arduino Uno) through which sensor data is transferred to Matlab on a personal computer via USB. With this implementation, sampling rates of approx. 62.5 Hz are achieved.

The built-in image sensor is a complementary metal-oxide semiconductor (CMOS) chip with dimensions of 30 × 30 pixels and a programmable sensitivity between 200 and 8200 cpi. Thus, the maximal resolution of the sensor calculates to approximately 3.1 × 10^−3^ mm ($\hat{=}$1/8200 cpi). This exceeds high-end motion capturing systems which have been used to evaluate interface dynamics [38].

Magnitude, velocity, and acceleration of relative movement between residual limb and prosthetic socket are expected to depend on gait dynamics and subsequent induced loads [20]. The maximal detectable velocity $v_{\max}$ and acceleration $a_{\max}$ of approximately 3810 mm/s, respectively 294,300 mm/s^2^ of the sensor exceed expected gait dynamics (v≤100 mm/s and a≤3000 mm/s^2^ cf. [20]).

Overall, the sensor unit specifications promoted in the data sheet satisfy the requirements set by the biomechanical measuring task. To quantify the true performance achieved by the realized sensor system, this paper assesses sensor functionality experimentally.

## 3. Experimental Evaluation on a Test Rig

This section assesses the functionality of the sensor unit on a test rig regarding variations of the measurement scenario, the sensor settings, and the environment. First, the descriptions of the generally valid methodology of the test rig assessment is presented. Two trial sessions differing in measurement factor variations are conducted. Section 3.2 and Section 3.3 include results and discussion of these sessions. Finally, a conclusion of the systematic sensor assessment on a test rig is given.

### 3.1. Methodology

**Figure 2:**
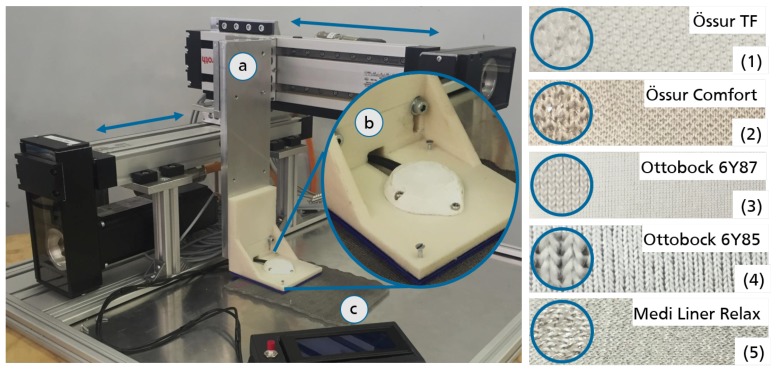
Test rig setup with indicated movement possibilities: **a** movable cantilever, **b** affixed sensor unit, and **c** stationary reference surface textures (**left**) and tested liner materials (**right**).

To generate defined, repeatable, and reliable movement trajectories, a two-axial test rig is used. It operates with a Rexroth MTX 13V programmable open-loop control system. Two linear drives (Indradyn, Bosch Rexroth, Lohr am Main, Germany) are used to move a cantilever over the base plate of the test rig. The sensor unit is affixed to the cantilever of the test rig while the reference surface texture (different prosthetic liner materials) is placed on its base plate (cf. Figure 2 left).

Each measurement trial consists of a certain number of repetitive movements of the test rig (test sequences). The relative movement generated by the test rig is not controllable in the intended biomechanical measurement scenario, hence test rig motion is used to quantify sensor functionality. Sensor performance is evaluated by consideration of the relative error $err_{rel}$:

(1) $$err_{rel}=\frac{(x_{1}-x_{0})-\Delta x}{\Delta x}$$

where Δx stands for known test rig motion, while $x_{1}$ and $x_{0}$ mark sensor position at the beginning, respectively end, of a test sequence.

**Figure 3:**
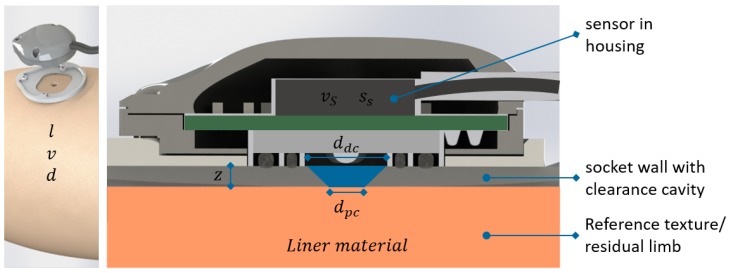
Illustration of factors and their influencing domain: the measuring task defines the factors distance *l*, velocity *v* and direction *d* (**left**). Calibration velocity $v_{s}$ and sensor sensitivity $s_{s}$ can be adjusted in the sensor settings. The thickness of the socket wall leads to the measuring distance *z*, the clearance cavity is defined by the proximal and distal diameter $d_{pc}$ respectively $d_{dc}$. Different liner materials serve as reference texture.

Additional to test rig movement variations, factors which supposedly influence sensor performance are varied: reference surface texture (The dependency of device functionality on reference surface texture has been discussed in several publications e.g., [33,39]), sensor settings (calibration velocity and sensitivity), and measurement environment (cavity diameter dimensions and measurement distance). To reduce the number of necessary trials, a two-stage fractional factorial screening design is used to evaluate sensor functionality considering the mentioned factors in two sessions. Figure 3 illustrates the evaluated factors for both trial sessions and groups them according to the influencing domain.

In a first session, sensor performance on different liner materials is assessed to identify a favorable sensor-liner configuration. Additionally, five factors (distance, velocity, and direction of test rig movement as well as sensor calibration velocity and sensor sensitivity setting) are tested. Following the two-stage fractional factorial screening design with resolution *V*, 16 experimental trials for each of the tested liner materials (materials 1–5 cf. Figure 2 right) are conducted.

A second session is realized with the identified favorable sensor-liner configuration and sensor settings from the first session. Six factors are examined: in addition to variations in test rig distance, velocity, and direction, measuring distance as well as upper and lower cavity diameter are varied. Corresponding to the two-stage fractional screening design with resolution *V*, 32 experimental trials for the most promising sensor-liner configuration of session one is completed.

The influence of a factor on the system behavior is called *effect* and can be visualized in the effect diagram. Main effects of a factor $E_{f}$ are represented as effect lines in the diagram by considering the mean value of all trials *n* comprising the same level (±) of the factor *f*:

(2) $$E_{f}=\frac{\sum_{i=1}^{n}err_{rel}\left(f_{i^{+}}\right)}{n}-\frac{\sum_{i=1}^{n}err_{rel}\left(f_{i^{-}}\right)}{n}.$$

Hence a level main effect line suggests an irrelevance of the factor on the sensor’s performance while a positive/negative gradient indicates one factor state to be beneficial for the sensor performance.

To identify suitable sensor settings and sensor-liner configurations, the effects of measurement variations on sensor performance are considered. Data evaluated according to Equation (1) is expected to be distributed around a mean close to zero for factor variations. To assess dependencies on sensor performance, data is analyzed via:

(3) $$err_{rel}^{\prime }=\frac{\left|(x_{1}-x_{0})-\Delta x\right|}{\Delta x}.$$

This shifts negative values of $err_{rel}$ vertically into the positive area. Thus, elevating the mean corresponding to the uncertainty of the data, enabling to differentiate between sensor performances for $err_{rel}^{\prime }$.

### 3.2. Trial Session 1: Sensor-Liner Configuration and Sensor Settings

In the first trial session, different sensor-liner configurations are evaluated with the aim to identify suitable combinations for the biomechanical measurement task. Additionally, the effects of variations concerning test rig movement, sensor calibration velocity, and sensor sensitivity are evaluated. Applied factor and parameter values are summarized in Table 1. The two-stage factorial fractional screening design with a resolution *V* is used for all five tested liner materials.

The two levels of the test rig movement factors are set by considering the biomechanics at the interface: relative movements can be as little as 5 mm while the maximum gradient can be estimated to 71 mm/s [20]. To account for the most challenging measurement situations, the values for minimal distance is set to 1 mm while the maximal velocity is set to 100 mm/s. The corresponding limits for the two factors are set to 10 mm and 1 mm/s. For variations in direction, the sensor’s main axes *x* and *y* are considered. Calibration is assumed to deliver best sensor functionality when matching the subsequently investigated measurement setting. Hence, the two used test rig velocities are chosen for calibration. Sensor sensitivity is varied between the upper and lower programmable limits: 200 and 8200 cpi. Measuring distance is appointed to the optimal setting corresponding to the sensor’s data sheet. To avoid interference with sensor functionality, cavity diameters are set to the outer dimensions of the used lens. One measurement trial (constant factor setting according to factorial design) consists of 10 test sequences for trial session 1.

#### 3.2.1. Results and Discussion

**Figure 4:**
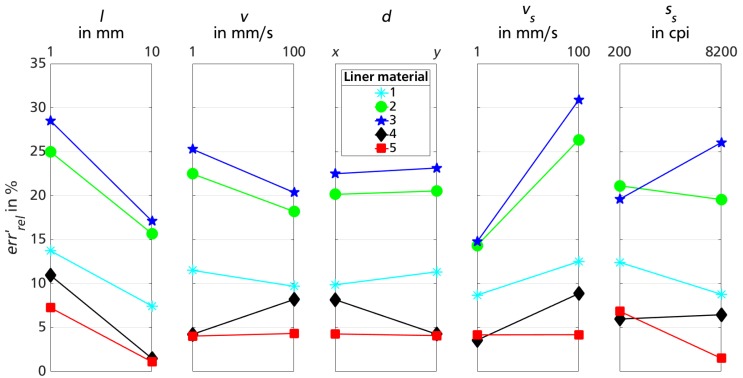
Effect lines for factor variation for measurements of trial session 1 on different liner materials 1–5.

Figure 4 displays the main effect lines for each varied factor for the different liner materials. In each subplot, the information of all conducted test sequences (160) is reduced to the mean $err_{rel}^{\prime }$ for reasons of clarity and comprehensibility.

The value of $err_{rel}^{\prime }$ ranges between 1.1% and 30.9%, largely depending on sensor-liner configuration. Liner materials 1, 4 and 5 show smaller mean relative errors over all presented data points compared to materials 2 and 3.

The effects of test rig movement variation also depend on the sensor-liner configuration. While the effect line gradient for motion distance $E_{l1}$ is negative for all liner materials, the algebraic sign of effect lines for velocity $E_{v1}$ and direction $E_{d1}$ are contrary for liner material 4 compared to materials 1–3. Sensor-liner configuration 5 shows a similar behavior for varied test rig motion velocity and direction ($E_{v1}\leq E_{d1}\leq 0.3$).

The effect line gradient of the factor sensor calibration velocity is positive for all liner materials, even though only slightly for material 5 ($E_{vs1}\leq 0.02$). The effect of sensor sensitivity once again depends on sensor-liner configuration: liner materials 1, 2 and 5 show a negative gradient, while sensor functionality on materials 3 and 4 demonstrate better results for lower sensitivity.

The first three plots of Figure 4 show variations on test rig movements, which are not controllable in the later biomechanical measurement scenario. Therefore, a level effect line would indicate an advantageous sensor-liner configuration. Generally, a tendency of $err_{rel}^{\prime }$ improving for larger distances can be observed. For smaller travel distances, the limited sensor resolution becomes more consequential. For variations in test rig velocity and traveling direction, the effect line gradient depends on sensor-liner configuration and, thus, liner material 5 shows the favorable robust behavior. While the calibration velocity $v_{s}$ shows almost no effect on sensor-liner configuration 5, a higher sensor sensitivity $s_{s}$ seems to be beneficial.

**Figure 5:**
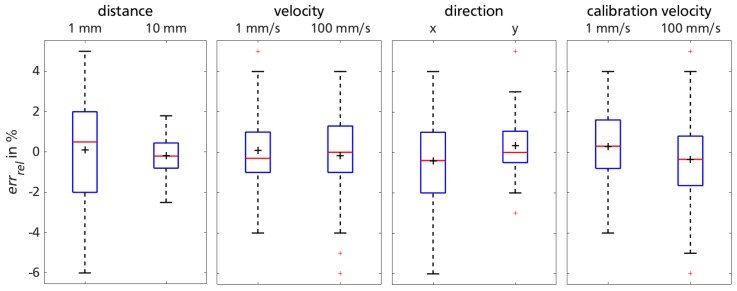
Sensor performance on material 5 for favorable high sensor sensitivity, grouped for remaining measurement factors of trial session 1.

Within the used fractional screening design, each two-factor interaction is confounded with three-factor or third-order interactions. Instead of displaying the confounding effects, Figure 5 displays the distribution of $err_{rel}$ for the most promising sensor-liner configuration: high sensor sensitivity on liner material 5. Results are visualized with grouped box plots, also yielding information about influencing factors concerning measurement variations.

The previously discussed dependency of sensor functionality on test rig distance is still apparent when considering the uncertainty of the data. The same applies to test rig direction which has been reported previously [35]. Test-rig velocity does not influence the sensor functionality significantly. Neither does the variation of sensor calibration velocity. Nevertheless, due to existing outliers for $v_{s^{+}}$ (cf. Figure 5, rightmost box plot), the slower calibration velocity will be used for further sensor functionality assessments.

#### 3.2.2. Implication

Excluding outliers, $err_{rel}$ does not reach a value above ±6% for any of the 80 measurement sequences represented in the box plots of Figure 5. Mean and standard deviation for data corresponding to the high sensor sensitivity setting ($s_{s+}=8200$ cpi) as well as low calibration velocity ($v_{s-}$ = 1 mm/s) calculates to $err_{rel,1}=$ 0.28 ± 1.73%. Hence, the favored sensor-liner configuration 5 is promising for practical application of measuring biomechanical interactions.

### 3.3. Trial Session 2: Measuring Distance and Cavity Diameter Variations

The second trial session considers the effect of variations of test rig movement, measuring distance as well as cavity diameter dimensions on sensor performance. The applied factor and parameter values are summarized in Table 2. Calibration velocity and sensor sensitivity are set to the previously established settings (cf. Section 3.2.2). The two levels for measuring distance variation are set to the operating range specified in the data sheet of the sensor. While the upper level of cavity diameter dimension is again set to the outer dimensions of the used lens, the lower level is set to 4.00 mm which corresponds to a common cavity dimension used in computer mice.

Measurement trials of session 2 are only performed on the favorable liner material 5. One measurement trial (constant factor setting according to factorial design) consists of 20 test sequences for this trial session.

#### 3.3.1. Results and Discussion

**Figure 6:**
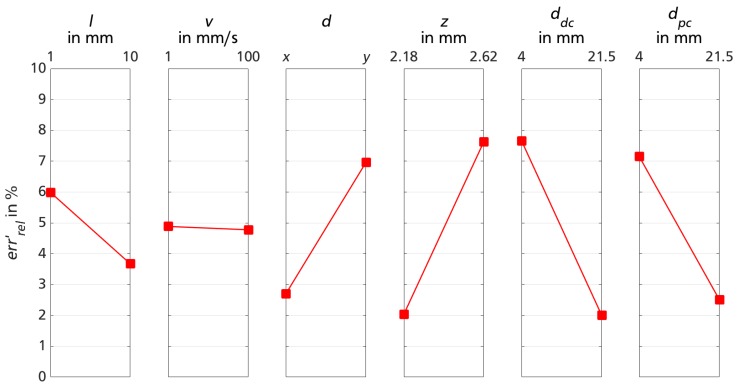
Effect lines for factor variation for measurements of trial session 2 on liner material 5.

Figure 6 shows the main effect lines of trial session 2 for each varied factor. In each subplot, the information of all conducted test sequences (640) is reduced to the mean of $err_{rel}^{\prime }$ for reasons of clarity and comprehensibility.

The effects of test rig movement for distance $E_{l2}=-2.32$ and velocity $E_{v2}=-0.11$ are comparable to those in trial session 1 ($E_{l1}=-6.17$ respectively, $E_{v1}=0.29$). Even though for session 2, both factors have a weaker influence on sensor functionality. The former little effect of traveling direction in favor of *y* ($E_{d1}=-0.19$) is now more relevant and shows lower errors in *x*-direction ($E_{d2}=4.27$). The effects of all varied factors of session 2 have an impact comparable to the one of sensor sensitivity in session 1. To quantify the influence of factor variations in trial session 2, a Wilcoxon rank sum test is used. Data collected with the deficient settings ($z_{+}\;,\;d_{dc-}\;\mathrm{and}\;d_{pd-}$) lead to significantly higher relative errors ($p_{1,z+}=0.0014,\;p_{1,ddc-}=1.8\times 10^{-4}\;\mathrm{and}\;p_{1,dpc-}=0.02$) of the sensor compared to the data with favorable settings of trial session 1. Contrarily to the cavity diameter dimensions, the distance between prosthetic socket and lower limb might vary in the later biomechanical measuring task. Thus, the impact of measuring distance *z* needs to be assessed further. The favorable settings for trial session 2 are narrowed down to $d_{dc+}=d_{pc+}=21.5\;\mathrm{mm}$.

**Figure 7:**
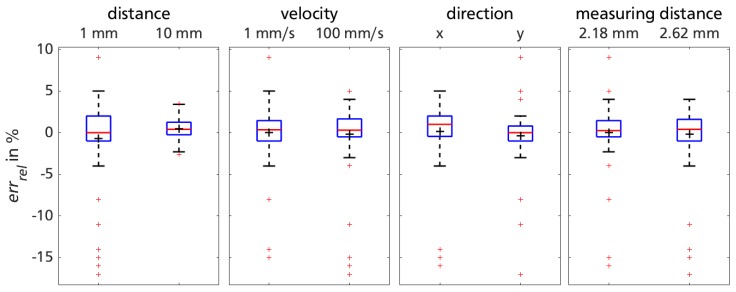
Sensor performance with favorable settings regarding cavity diameter dimension, grouped for remaining measurement factors of trial session 2.

Figure 7 displays the results obtained in the 160 test sequences performed with the mentioned favorable sensor settings ($d_{dc+}=d_{pc+}=21.5\;\mathrm{mm}$).

Data captured under the positive cavity diameter dimensions ($d_{dc+}=d_{pc+}=21.5$ mm) show smaller effects for remaining measurement factors: differences in means for $d_{-}$ and $d_{+}$ as well as $z_{-}$ and $z_{+}$ are reduced drastically compared to Figure 6. Neglecting outliers, $err_{rel}$ does not reach values outside ±5%, leading to similar results compared to trial session 1. Outliers are more prominent for shorter distances $l_{-}$ as can be seen in the leftmost box plot in Figure 7. The analysis of outlier occurrences ($err_{rel}\left(l_{-}\right)\leq -5$%) reveals a systematic error: all originate from the first test sequence of the repetitive movements of the test rig. Comparing data of the first test sequences of each trial with the remaining data affirms a significant difference in $err_{rel}$ (Wilcoxon rank sum test $p_{\mathrm{outlier}}=3.7904\times 10^{-6}$). For smaller test rig motion, the sensor systematically underestimates the traveling distance *l* in the first test sequence.

#### 3.3.2. Implication

Excluding the identified data of the erroneous first test sequence of each trial, mean and standard deviation of the remaining 152 test sequences with the favorable cavity diameter dimensions calculates to $err_{rel,2}=0.59\pm 1.79$%. Comparing results to trial session 1 ($err_{rel,1}=0.28\pm 1.73$%) suggest a relatively modest impact of small measuring distance variations z=2.4±0.22 mm.

### 3.4. Conclusion

The systematic assessment of sensor functionality on a test rig shows the general suitability of the sensor for the proposed measuring task. In trial session 1, an applicable reference surface as well as favorable sensor settings could be identified. Trial session 2 revealed the benefit of a larger diameter dimension for the upper and lower rim of the cavity compared to the one commonly used in computer mice. Additionally, a systematic error of the sensor for the first test sequence in short test rig movements could be identified.

With a calibration velocity of 1 mm/s, a sensor sensitivity of 8200 cpi and cavity diameter dimensions of 21.5 mm, the sensor showed appropriate functionality with a relative error of $err_{rel}=0.52\pm 1.78$% on liner material 5 (Medi Liner Relax (Bayreuth, Germany)) in overall 192 test sequences with varied test rig motion (distance l=1 respectively 10 mm, velocity v=1 respectively 100 mm/s, direction) and measuring distance (z=2.4±0.22 mm).

The experimental evaluation on the test rig as proposed in this section has the advantage of testing sensor functionality for defined, repeatable, and reliable movement trajectories. Nevertheless, it has certain limitations when it comes to mimicking the biomechanical measurement task. For instance, for test rig assessment the reference texture (liner material) is affixed to the base plate of the test rig., restricting the even measurement surface to remain stationary during measurements. In the biomechanical environment, the lower limb serves as the measurement surface for the sensor, which poses the challenge of measuring on a convex area that moves dynamically relative to the also moving prosthetic socket.

## 4. Experimental Evaluation during Gait Using an Orthosis

This section aims at estimating the effect of the above-mentioned challenges on the measurement quality of the sensor. An orthosis is used to experimentally evaluate the sensor suitability for detecting relative movement during human gait. Two healthy participants (male, partic. 1: 25 years old, 169 cm, 64 kg and partic. 2: 24 years old, 200 cm, 87 kg) agreed to take part in the experimental evaluation. Except for cavity diameter dimensions, the favorable sensor settings and liner material of the previously described test rig assessment are used for the measurement tasks ($d_{dc}=d_{pc}=4$ mm, $v_{s}=1$ mm/s, $s_{s}=8200$ cpi, z = 2.4 ± 0.22 mm, and liner material 5). The sensor functionality is assessed for two different measurement tasks: unloaded knee bends and treadmill gait. The two chosen tasks differ in controllability, dynamics, and observability. While disturbance variables can be minimized and sensor functionality is quantifiable for unloaded knee bends, treadmill gait imitates the intended biomechanical measurement task and environment with all the related complications.

In this section, sensor functionality for both measurement tasks is evaluated. The two Section 4.1 and Section 4.2 present the used methodology and discuss the results concerning sensor functionality. Finally, a conclusion regarding the suitability of the sensor for measuring the relative movement between residual limb and prosthetic socket during dynamic gait situations is drawn.

### 4.1. Unloaded Knee Bends

Data gathered during unloaded knee bends can be used to assess sensor functionality on a convex human measurement surface: the sensor moves along the proximodistal (pd) axis of the shin. Sensor movement is induced through the performance of unloaded knee bends. Compared to the biomechanical measurement task, disturbance variables are minimized. Due to little muscle activity around the measurement surface, liner material elongation and changes in measuring distance *z* are reduced.

#### 4.1.1. Methodology

Unloaded knee bends are performed at two different metronome-defined frequencies (0.25 Hz respectively 0.38 Hz). A set of five unloaded bends is performed by sitting on a chair and moving the foot between two marks on the floor. The gap between the two marks is set according to the participant’s foot length: one mark is at the posterior end of the foot when sitting with a 90° flexed knee, the second mark is set two foot lengths apart at the front side of the foot.

**Figure 8:**
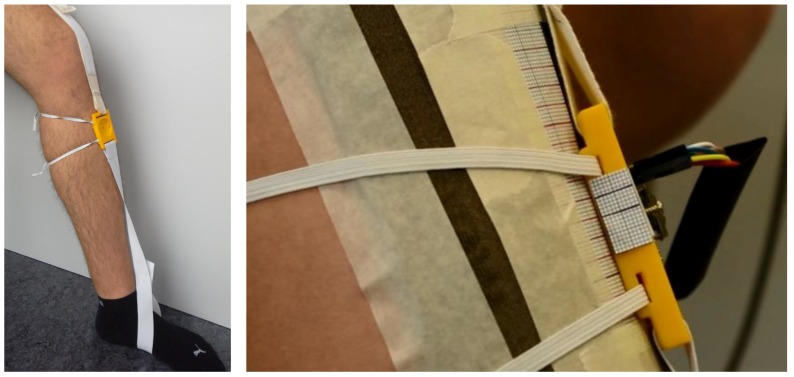
Measurement setup to assess sensor functionality during unloaded knee bends: sensor platform with elastic attachment (**left**) and integration of quantification method (**right**).

The sensor is attached elastically between foot and thigh, performing a movement along the pd-axis of the shank. A patch of liner material 5 is affixed to the shin, serving as measurement surface for the sensor. To facilitate a quantification of the sensor performance, scaled paper is attached to the shank and to the sensor (cf. Figure 8). In addition to sensor data acquisition, the movement along the scaled paper is recorded with 25 fps using a Pentax K5 camera and Pentax SMC DA 18–55mm F3.5–5.6 AL WR lens (K.K. Ricoh, Tokyo, Japan).

Data is evaluated offline via manual visual read of the scaled paper. To meet the uncertainty of the scaled paper reading (spr), an error bar of δspr=±1 mm is introduced. Due to a missing trigger signal between camera and sensor data, data needs to be synchronized offline. The offset calculation is handled via fitting of the data in the reversal points of the repetitive movement (knee bends): Maxima and minima evaluation in both data sets and shifting spr-data according to the calculated mean offsets of both data comparison points. Uncertainty in time recognition due to incorrect camera frame assessment and continued maxima registration of sensor (0.1 s) calculates to

(4) $$\delta t=\sqrt{{\left(\frac{1}{25}\;s\right)}^{2}+{\left(0.1\;s\right)}^{2}}=0.11\;s,$$

following the Gaussian propagation of uncertainty. Thus, an error bar of δt=±0.11 s is introduced.

#### 4.1.2. Results and Discussion

**Figure 9:**
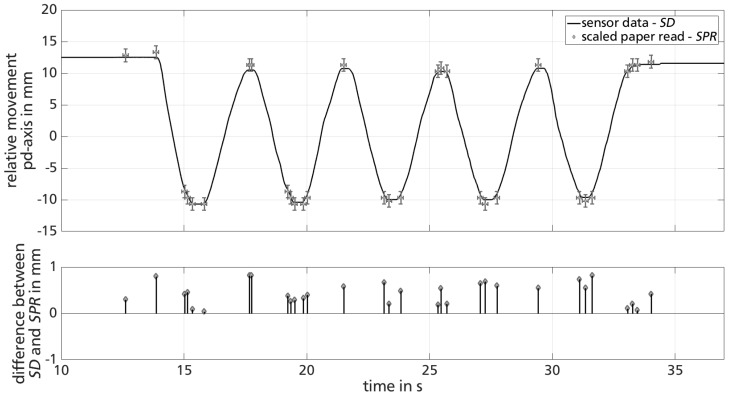
Representative plot of sensor data and scaled paper read (**top**) and calculated difference (**bottom**) of partic. 2 at 0.25 Hz.

The results of one set of unloaded knee bends (partic. 1 at 0.25 Hz) are representatively shown in Figure 9 (top). Sensor data is sampled with an averaged frame rate of 62.5 Hz and plotted continuously, while scaled paper readings are analyzed in discrete time instants, mainly within the turning points of the unloaded knee bends. The proposed two-dimensional error bars for time δt=±0.11 s and scaled paper readings δspr=±1 mm are plotted for each discrete scaled paper read.

After remaining at a constant position, the sensor registers five repetitive movements of around 23 mm (12<t<35) before remaining stationary again. These five repetitions match the number of knee bends in one set. Figure 9 (bottom) shows the calculated difference between sensor data sd and scaled paper readings spr at the discrete evaluation points. Data differences remain within the introduced ±1 mm error bar of spr (cf. Figure 9 (bottom)). Nevertheless, the mean and standard deviation of differences between sd and spr ($\bar{x}\pm \sigma x$) of 0.45±0.25 mm suggest a slight but systematic error within sensor data sd.

Table 3 summarizes the results for both participants at the two metronome-defined frequencies. To account for statistical evaluation, the number of evaluation points of spr is also noted. Additionally, mean and standard deviation as well as maximum relative error $\max(err_{rel}$) of each data set is included. $err_{rel}$ is calculated analogously to Section 3.

Due to different foot lengths, the induced movement along the shank Δx varies among participants (approximate 15 mm for partic. 1 respectively 24 mm for partic. 2), leading to smaller relative errors for participant 2 for similar absolute error values. The previously mentioned slight systematic error is apparent for all evaluated data sets: $\bar{x}-\sigma x\geq \;0.1$ mm (cf. column four in Table 3). Mean and standard deviation of the absolute error are similar for all test settings: there is no tendency of absolute errors $err_{abs}$ increasing with knee bend frequency or absolute errors generally being higher for either participant.

Even though slightly underestimating the relative movements, the sensor functionality on a convex surface is satisfactory. With absolute errors $err_{ab}$ ≤ 1 mm, the sensor fulfills the requirements set in [29].

### 4.2. Treadmill Gait

An orthosis is used to imitate the proposed measurement task to evaluate the suitability of the sensor. With the established experimental setup, a gait-induced motion similar to the expected relative movement between residual limb and prosthetic socket during gait (cf. [20]) is achieved.

#### 4.2.1. Methodology

**Figure 10:**
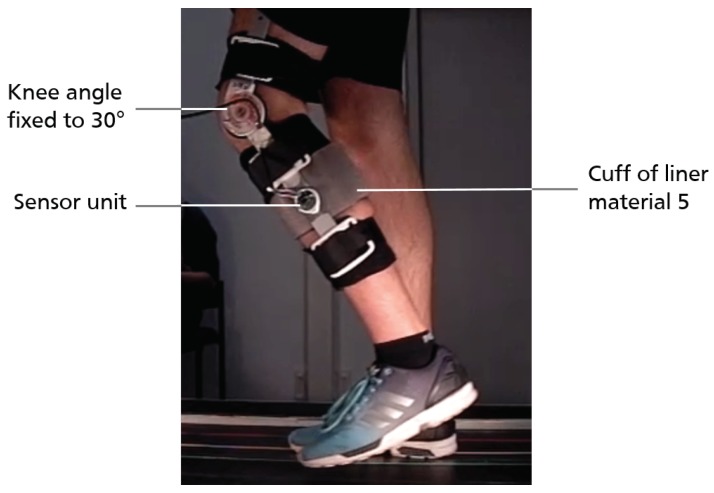
Measurement setup for treadmill gait: the sensor unit is attached to the lateral splint of the orthosis, measuring the relative movement between shank (cuff of liner material 5) and orthosis.

To ensure a gait-induced relative movement of the shank within the knee orthosis, the knee joint of the orthosis is locked at 30° flexion. Each participant performs around 100 gait cycles at three different treadmill velocities ($v_{tm}=1\;\pm$ 0.4 m/s). The sensor is attached on the lateral splint of the orthosis, detecting movements in the sagittal plane of the shank within the orthosis (cf. Figure 10). Participants wear a cuff of liner material 5 which serves as the measuring surface for the sensor. Due to the inner silicone layer of the liner, relative movement between shank and liner material is assumed to be negligible for the limited time of data acquisition.

Sensor data acquisition continues during the completion of the approximately 100 gait cycles on the treadmill. In contrast to the unloaded knee bends, this measuring task is more dynamic and movements are expected to be of smaller magnitude. Accordingly, the relative movement between orthosis and the lower limb is not observable with the proposed camera-scaled paper read method of the previous section.

Instead, sensor data is evaluated via a biomechanical plausibility check. Sensor data should show a repetitive pattern over the measurement period, reflecting the gait-induced relative movement. The sensor signal is segmented automatically according to the identified peaks in the signal (Findpeaks-funtion in Matlab (MathWorks, Natick, MA, USA)). Sensor data segments do not necessarily have the same length, which is due to the variability of gait. For mean and standard deviation calculations, segment lengths are interpolated to the same number of data points. Additionally, the recorded data of each trial is analyzed via fast Fourier transformation (FFT). Studies [40,41] show how humans adjust their gait with speed. Common strategies are enlargement of stride length as well as higher cadence with increasing gait velocity. To ensure foot clearance, swing phase knee flexion has a fairly straightforward relationship to stride length [40]. Accordingly, sensor data is evaluated concerning changes in main frequency as well as peak-to-peak values with variation of treadmill speed. Since relative movement is induced by a fixed knee angle, the means of resulting segmented sensor signals are discussed in comparison to ordinary knee angle movement trajectories during gait.

#### 4.2.2. Results and Discussion

**Figure 11:**
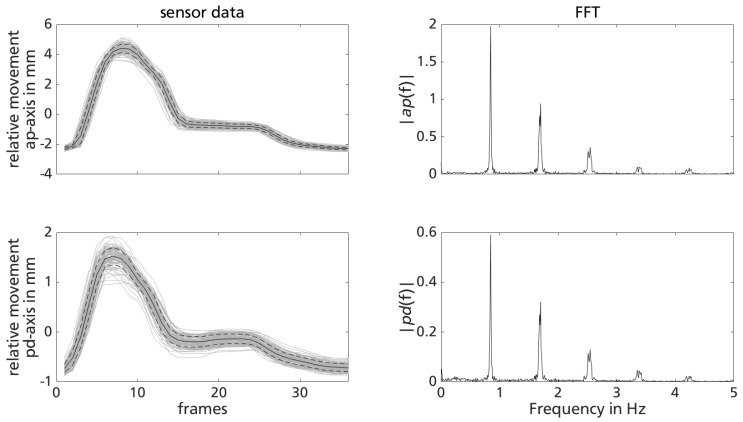
Representative plot for sensor data evaluation: mean ± standard deviation (black lines) of identified repetitive pattern in sensor data for both directions (**left**) as well as results of FFT (**right**) for trial session of partic. 1 with $v_{\mathrm{tm}m}$.

Results of sensor data analysis (segmented sensor data signal for both directions as well as FFT) look similar for all six treadmill gait trials. Representatively, the results of the trial session with medium gait velocity $v_{\mathrm{tm}m}$ of participant 1 are shown in Figure 11. The left column includes the segmented sensor signal for the sensor’s main axes: 97 signal segments (grey) as well as calculated mean (black) ± standard deviation (black dashed) are depicted. The right column contains the results of the FFT analysis of the entire sensor data signal for the trial session.

Segmented sensor signals show qualitatively the same curve shape. While peak-to-peak values in anteroposterior (ap) direction calculate to 6.74±0.19 mm, movement in pd-direction measures to 2.28±0.21 mm. The variability within segmented sensor signals appears to be similar to the gait variability of humans. The main frequency of the sensor signal for both directions calculates to 0.85 Hz. Further peaks in the FFT represent the harmonics (i.e., multiples).

The results of the treadmill gait trials for the two participants are summarized in Table 4. The number of identified gait cycles in the sensor signal $n_{\mathrm{Gait}}$, mean and standard deviation of peak-to-peak relative movement for both sensor directions as well as first harmonic of FFT of the sensor data (registered cadence of gait $f_{\mathrm{Gait}}$) are given.

With increasing gait velocity, sensor data for participant 1 registers greater movements in ap as well as pd-direction. The Wilcoxon rank sum test shows a statistical significance between peak-to-peak values across different treadmill velocities for both directions. *p*-Values ranging between 1.36 × 10^−34^ (ap-direction $v_{2}$ and $v_{3}$) and 1.12 × 10^−27^ (pd-direction $v_{1}$ and $v_{2}$). Additionally, the FFT of the signal data indicate an increasing cadence with higher treadmill velocity.

The analysis of the sensor data gathered with participant 2 also shows increasing peak-to-peak values with higher treadmill velocity. The Wilkoxon rank sum test reveals statistical significance between data of different treadmill velocities ranging from 2.01 × 10^−35^ (pd-direction $v_{2}$ and $v_{3}$) and 1.16 × 10^−25^ (pd-direction $v_{1}$ and $v_{2}$). The FFT analysis also suggests an increasing cadence with higher treadmill velocity.

Analysis of sensor data indicates that both participants achieve walking with treadmill speed through adaptation of cadence as well as step length. Thus, sensor data seems to deliver biomechanically plausible results. Nevertheless, while results of the FFT are similar for participant 1 and 2, relative movement is significantly greater for participant 1 in both sensor directions ($1.78\times 10^{-35}\leq p_{ap}\leq 1.15\times 10^{-32}$, respectively $1.78\times 10^{-35}\leq p_{pd}\leq 2.47\times 10^{-33}$). This interpersonal difference is not examined further, but might be explicable through anthropometric dissimilarity of the two participants as well as individual adaptation strategies to the biomechanical disturbance of the fixed knee angle.

**Figure 12:**
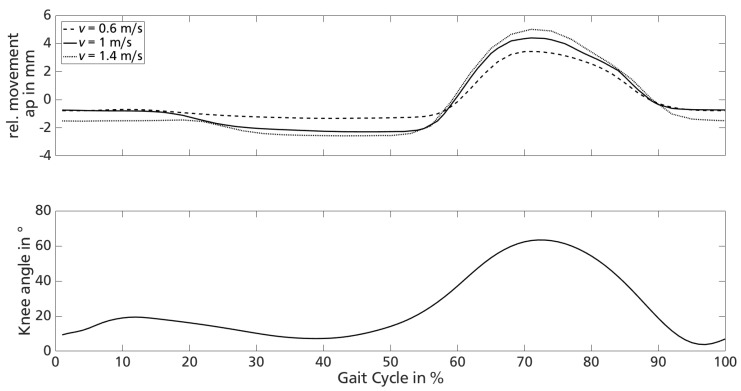
Comparison of shifted sensor data mean (**top**) and changes in knee angle over gait cycle according to (**bottom**).

Figure 12 compares the mean of sensor data in ap-direction of participant 1 at different treadmill velocities to the knee angle movement over one gait cycle according to available data taken from [43]. The mean of sensor data in ap-direction is shifted along the time axis to adjust the time axis of sensor data to the presentation of the gait cycle. The analysis of video data substantiates the phase of greatest relative movement to be during swing phase knee flexion.

The acquired movement data shown in Figure 12 (top) correlate to the velocity-dependent increase of knee extension (approximate 40% of the gait cycle) and flexion (approximate 70% of the gait cycle), matching reported increasing knee flexion as well as extension peak with self selected slow, medium and fast walking speed in 40 healthy individuals [44].

The analysis of the recorded sensor data shows rising main frequencies of the signal as well as increasing peak-to-peak values with higher gait velocities. The segmented relative movement can be assigned to knee angle motion during a gait cycle. Compared to literature results, the sensor delivers biomechanically plausible data.

### 4.3. Implication

For unloaded knee bends, the induced relative movement (15 mm respectively 24 mm) exceeds the one expected between residual limb and socket in dynamic gait situations. Nevertheless, in contrast to test rig evaluation, the supervised task of unloaded knee bends poses the challenge for the sensor to measure movement on the shank: a convex surface. Additionally, both objects (sensor and shank) are in motion during unloaded knee bends. The presented results show the fulfillment of the sensor accuracy compared to previously formulated requirements ($err_{abs}\leq 1$ mm) [29], which underlines the suitability of the sensor for the proposed measurement task.

As sensor functionality is not quantifiable during treadmill trials, a biomechanical plausibility check is performed. It reveals that the sensor data is meaningful since peak-to-peak movement and cadence increase with walking speed. Additionally, curve shapes of segmented sensor data are similar for all test trials and comparable to changes of knee angle over a gait cycle. The detected range of motion is similar to the expected relative movement in the planned measuring task.

The combination of results for the different scenarios indicates the feasibility of measuring the relative movement between residual limb and prosthetic socket in dynamic gait situations with a measurement system based on the assessed sensor unit.

## 5. Conclusions

The systematic experimental assessment of the proposed sensor system based on a 2D-motion sensor revealed the suitability of the sensor unit for measuring the relative movement between outer liner surface (residual limb) and prosthetic socket in dynamic gait situations.

Through test rig evaluation, an advantageous sensor-liner configuration is identified. Using a two-factorial fractional screening design, sensor functionality dependence on calibration velocity, sensor sensitivity, measuring distance and cavity diameter dimensions are assessed and favorable settings are established. Considering these favorable settings (calibration velocity of 1 mm/s, sensor sensitivity of 8200 cpi, and cavity diameter dimensions of 21.5 mm), sensor functionality is quantified to $err_{rel}\;=\;0.52\;\pm \;1.78$% for relevant testing scenarios.

Advancing to the measurement task of unloaded knee bends facilitates the investigation of sensor performance on convex surface, which is closer to the final application. Data of this dynamic, yet observable measurement task, revealed absolute sensor errors of $err_{abs}\leq 1$ mm. Finally, the feasibility of measuring gait-induced relative movement with the proposed 2D-motion sensor is shown: the sensor delivers biomechanically plausible results for straight level walking on a treadmill at different gait velocities.

Thus, the experimental evaluation of the sensor system discussed in this paper substantiates the suitability of the sensor for the biomechanical measurement task. Future work will include the realization of the socket measurement system concept proposed in previous work [29] and testing the system in a pilot study with a unilateral transtibial amputee using a PTB socket with shuttle lock suspension. In order to conduct experimental trials with participants using a different type of suspension systems, the effectiveness of the proposed sensor seals need to be evaluated. The identification of the individual coupling stiffness through measurement of relative movement will help to understand the dynamic interactions at the residual limb-socket interface and might lead to the improvement of socket designs.

## Figures and Tables

**Table 1:** Values of two-stage fractional factorial screening test factors and parameters for trial session one.

Factor/Parameter	Abbreviation	Two-Stage Factorial Settings	
		−	+
Distance	*l*	1 mm	10 mm
Velocity	*v*	1 mm/s	100 mm/s
Direction	*d*	*x*	*y*
Sensor calibration velocity	$v_{s}$	1 mm/s	100 mm/s
Sensor sensitivity	$s_{s}$	200 cpi	8200 cpi
Measuring distance	*z*	2.4 mm	
Cavity diameter (distal)	$d_{dc}$	21.5 mm	
Cavity diameter (proximal)	$d_{pc}$	21.5 mm	

**Table 2:** Values of two-stage fractional factorial screening test factors and parameters for trial session two.

Factor/Parameter	Abbreviation	Two-Stage Factorial Settings	
		−	+
Distance	*l*	1 mm	10 mm
Velocity	*v*	1 mm/s	100 mm/s
Direction	*d*	*x*	*y*
Measuring distance	*z*	2.18 mm	2.62 mm
Cavity diameter (distal)	$d_{dc}$	4 mm	21.5 mm
Cavity diameter (proximal)	$d_{pc}$	4 mm	21.5 mm
Sensor calibration velocity	$v_{s}$	1 mm/s	
Sensor sensitivity	$s_{s}$	8200 cpi	

**Table 3:** Summarized results of unloaded knee bends task.

Data Set		Evaluation Points *n*	$\bar{x}\pm \sigma x$ in mm	*n* Outside δspr	Max(${\mathrm{err}}_{\mathrm{rel}}$)
1	partic. 1/0.25 Hz	58	0.34±0.24	-	5.73%
2	/0.38 Hz	51	0.43±0.29	-	6.47%
3	partic. 2/0.25 Hz	32	0.45±0.25	-	3.42%
4	/0.38 Hz	24	0.35±0.24	-	4.06%

**Table 4:** Summary of sensor data analysis for gait on treadmill of participant 1 and 2 (cf. [42]).

Gait Velocity		$n_{\mathrm{Gait}}$	Movement in		$f_{\mathrm{Gait}}$
			ap-Direction	pd-Direction	
partic. 1	0.6 m/s	93	(4.87 ± 0.40) mm	(1.62 ± 0.29) mm	0.68 Hz
	1.0 m/s	97	(6.74 ± 0.19) mm	(2.28 ± 0.21) mm	0.85 Hz
	1.4 m/s	104	(7.70 ± 0.19) mm	(2.85 ± 0.27) mm	0.96 Hz
partic. 2	0.6 m/s	97	(0.75 ± 0.17) mm	(0.19 ± 0.08) mm	0.69 Hz
	1.0 m/s	111	(1.17 ± 0.22) mm	(0.44 ± 0.14) mm	0.86 Hz
	1.4 m/s	99	(1.92 ± 0.26) mm	(1.22 ± 0.22) mm	0.97 Hz

## Abbreviations

SPI	Serial Peripheral Interface
PLA	Polylactide
USB	Universal Serial Bus
CMOS	Complementary metal-oxide-semiconductor
pd	proximodistal
ap	anteroposterior
spr	scaled paper reading
sd	sensor data
FFT	Fast Fourrier Transformation
